# Targeting Tie-2/angiopoietin axis in experimental mesothelioma confers differential responses and raises predictive implications

**DOI:** 10.18632/oncotarget.25004

**Published:** 2018-04-24

**Authors:** Sophia Magkouta, Apostolos Pappas, Ioannis S. Pateras, Androniki Kollintza, Charalampos Moschos, Maria-Eleni Vazakidou, Vasiliki Karavana, Vassilis G. Gorgoulis, Ioannis Kalomenidis

**Affiliations:** ^1^ Marianthi Simou Laboratory, 1st Department of Critical Care and Pulmonary Medicine, National and Kapodistrian University of Athens, School of Medicine, Evangelismos Hospital, Athens, 10675, Greece; ^2^ Molecular Carcinogenesis Group, Department of Histology and Embryology, School of Medicine, National Kapodistrian University of Athens, Athens, GR-11527, Greece; ^3^ Biomedical Research Foundation of the Academy of Athens, Athens, GR-11527, Greece; ^4^ Faculty of Biology, Medicine and Health, University of Manchester, Manchester Academic Health Science Centre, Manchester, M20 4QL, UK

**Keywords:** malignant pleural mesothelioma, angiopoietins, Tie-2, tumor angiogenesis, murine Tek-delta Fc

## Abstract

Malignant pleural mesothelioma is resistant to currently used treatment. Angiopoieitn-1 directly promotes mesothelioma cell growth in a Tie-2-dependent fashion. Angiopoietin/Tie-2 axis may thus be valid targets for therapeutic interventions against mesothelioma. We hypothesized that a soluble angiopoietin inhibitor (Murine Tek-deltaFc) would halt mesothelioma progression *in vivo* by enhancing mesothelioma cell proliferation and inhibiting tumor angiogenesis. Our hypothesis was challenged on two syngeneic mesothelioma *in vivo* models (AB1 cells-Balb/c mice and AE17 cells-C57BL/6 mice. Even though both mesothelioma cell lines express the Angiopoietin-1/-2 and Tie-2, murine Tek-deltaFc hampered AB1 but not AE17 mesothelioma growth *in vivo* by enhancing tumor cell apoptosis and limiting tumor angiogenesis. Neither angiopoietins (Angs)-1 and -2 nor the inhibitor affected mesothelioma cell growth *in vitro*. AB1 (responding) tumors were more vascularized and displayed higher endothelial Tie-2 and lower tumor Ang-1 expression than the (non-responding) AE17 tumors. Angiopoietins-1 and -2 are expressed in tumors and pleural cavity of mesothelioma patients demonstrating the clinical relevance of our experimental observations. In conclusion, disrupting Ang-Tie-2 signaling limits mesothelioma angiogenesis and halts tumor progression. Tumor vascularity, endothelial Tie-2 expression and tumor Ang-1 expression may predict mesothelioma response to Tek-deltaFc.

## INTRODUCTION

Malignant pleural mesothelioma (MPM) is an aggressive tumor of the pleural cavity, characterized by ominous prognosis. Since, the vast majority of cases result from asbestos exposure, a material that is still used in the most populous countries of the world, it is expected that a global epidemic of the disease will occur in the following decades [1]. Furthermore, experimental evidence suggests that modern artificial fibers may also be involved in mesothelioma development [2, 3].

No treatment has so far been substantially beneficial for patients with MPM [1]. Cytotoxic drugs, marginally prolong survival [4, 5, 6], radiotherapy is ineffective and the usefulness of surgery is strongly disputed [7]. In an effort to develop novel effective and safe therapeutic modalities, researchers are testing a wide variety of interventions, including anti-angiogenic treatment [8]. Akin to this, a combination of an anti-Vascular Endothelial Growth Factor (VEGF) antibody and standard chemotherapy has been recently shown to be more effective than chemotherapy alone [9].

Angiopoietins are also crucial for tumor angiogenesis and can be targeted to treat cancer [10]. Ang-1 to -4, exert their actions mainly through binding to a tyrosine kinase receptor (Tie2) which is principally expressed by endothelial cells [11]. Ang-2 mainly originates from the vascular endothelium and is responsible for vessel regression and destabilization and can enhance angiogenesis in a context-dependent manner [12, 13]. As for Ang-1 its major source is the peri-vascular cells. Ang-1 promotes vessel maturation and pericyte recruitment. Ang-1, Ang-2 and Tie-2 can be also expressed by tumor and inflammatory cells [14]. Little is known about the role of angiopoietins in MPM. Tabata et al, [15] showed that Ang-1 directly stimulates Tie-2 expressing mesothelioma cell proliferation and migration *in vitro* and that high serum Ang-1 levels are associated with shorter patient survival. Based on the above, we hypothesized that angiopoietin blockade would halt experimental mesothelioma progression *in vivo* by a) preventing a direct Ang-1-provoked growth effect on mesothelioma cells and b) inhibiting tumor angiogenesis. We tested our hypothesis using the angiopoietin inhibitor murine Tek-deltaFc (soluble extracellular domain of murine Tie-2 receptor that prevents binding of Ang-1 and Ang-2 with the naturally occurring receptor) on two syngeneic models of pleural mesothelioma [16].

## RESULTS

### AB1 and AE17 mesothelioma models display different basal angiogenic profiles

**Figure 1:**
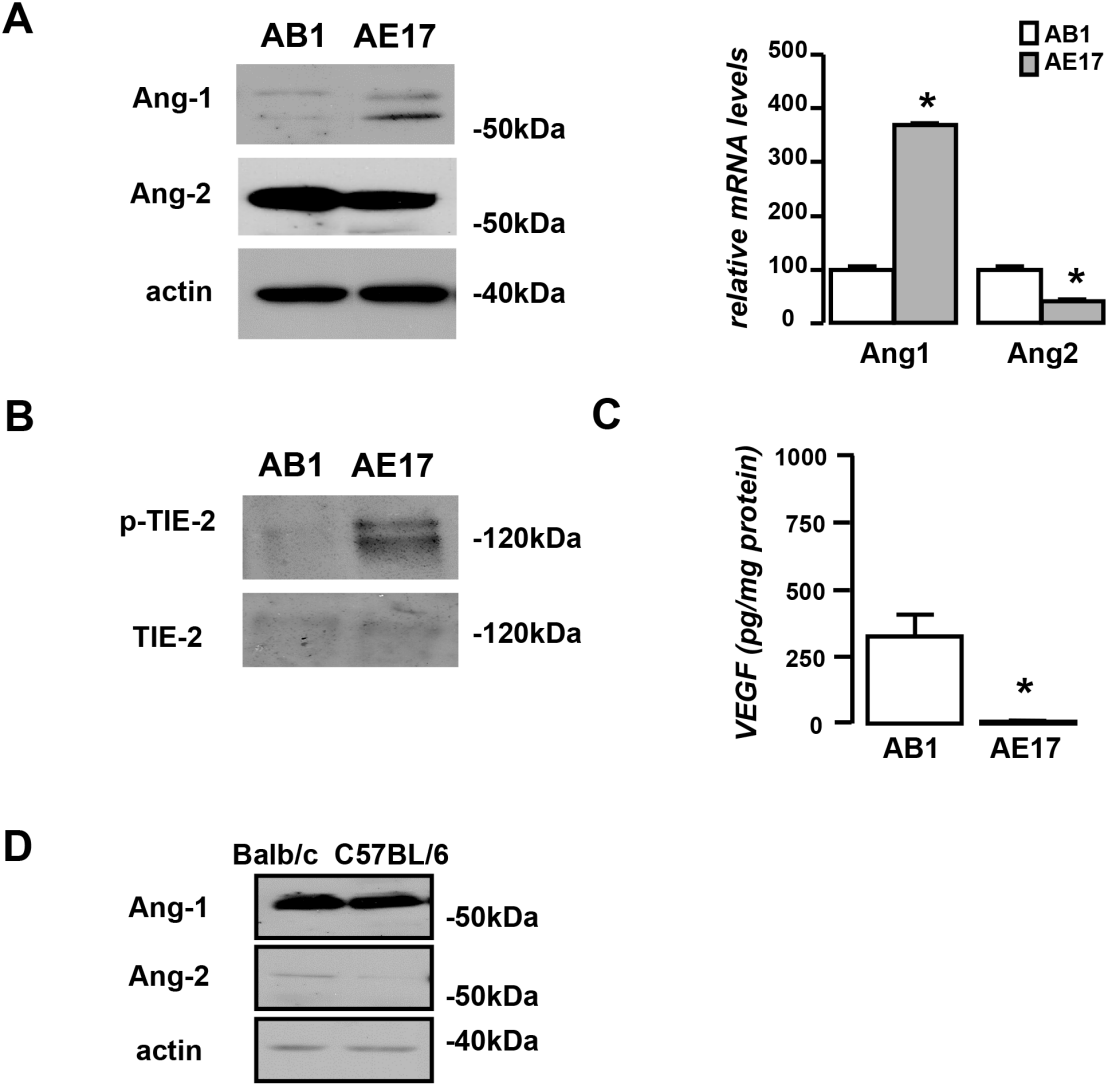
AB1 and AE17 mesothelioma cells present divergent angiogenic profiles **(A)** AB1 and AE17 mesothelioma cells were analyzed for Ang-1 and Ang-2 basal expression levels by western blot and Real-time PCR (left and right upper panels). Results were normalized to actin or GAPDH expression, respectively. **(B)** Basal Tie-2 activation status was evaluated in both cell lines using immunoprecipitation. **(C)** Basal VEGF secretion of both cell lines was also measured using ELISA. Data presented as mean±SEM, n=3-5, ^*^p<0.05 compared to AB1. **(D)** Balb/c and C57BL/6 normal pleural tissues were analyzed for Ang-1 and -2 expression.

**Figure 2:**
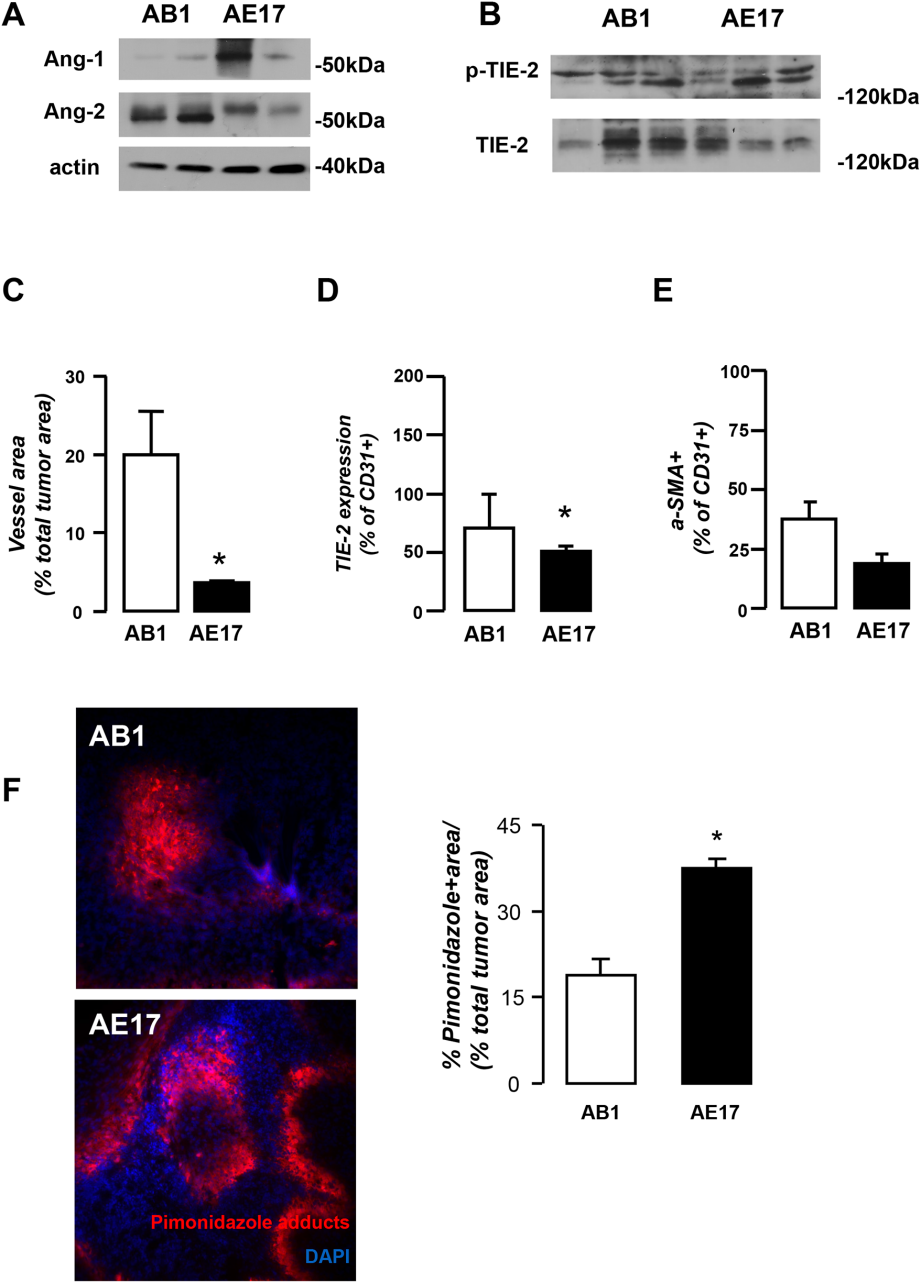
AB1 and AE17 mesothelioma mouse models present divergent angiogenic profiles **(A)** AB1 and AE17 pleural tumor tissues were analyzed for Ang-1 and -2 expression using western blot (A) as well as for activated (p-Tie-2) and total Tie-2 levels using immunoprecipitation **(B)**. Data presented as mean±SEM, n=5-6, ^*^p<0.05 compared to AB1. AB1 and AE17 tumors were analyzed for proportional vessel area **(C)**, endothelial Tie-2 expression **(D)** and proportional a-SMA+ vessels **(E)**. Data presented as mean±SEM, n=7-12, ^*^p<0.05 compared to AB1. **(F)** Immunofluoresent staining of pimonidazole adducts (left, Pimonidazole adducts: red, DAPI: blue) of AB1 and AE17 mesothelioma tumors and hypoxia quantification (right). Data presented as mean±SEM, n=5, ^*^p<0.05 compared to AB1.

We first characterized our experimental systems as for the expression and activation status of Angiopoietin/Tie-2 system and the closely related VEGF. AB1 cells were found to express more Ang-2 and less Ang-1 than AE17 cells (Figure 1A) and to secrete larger quantities of VEGF *in vitro* (Figure 1C). Tie-2 was expressed by both cell lines while it is more intensively activated in AE17 cells (Figure 1B). Regarding the expression of angiopoietins by the host pleural tissue, Ang-1 prevailed in both mouse strains, but Balb/c mice expressed higher levels of pleural Ang-2 compared to C57BL animals (Figure 1D). To examine whether this profile is maintained *in vivo*, tumor lysates of both models were compared in terms of the aforementioned angiogenic factors. In accordance to the *in vitro* findings, Ang-2 was more abundant in AB1 than AE17 tumors which, on turn, contained higher levels of Ang-1 (Figure 2A). AB1 and AE17 tumor lysates contained equal levels of total and activated Tie-2 (Figure 2B). AB1 tumors were more vascular (Figure 2C), less hypoxic (Figure 2F) and displayed higher levels of endothelial Tie-2 expression (Figure 2D) compared to AE17 ones. Pericyte coverage of tumor vessels did not significantly differ between the two models (Figure 2E). Overall, the Balbc-AB1 mesothelioma model is characterized by elevated host- and tumor-derived Ang-2, lower Ang-1 tumor content and increased vascularity and higher Tie-2 expression by endothelial cells compared to the C57BL-AE17 model.

### MuTekDeltaFc abrogates AB1 mesothelioma progression by inducing AB1 cell apoptosis *in vivo*

**Figure 3:**
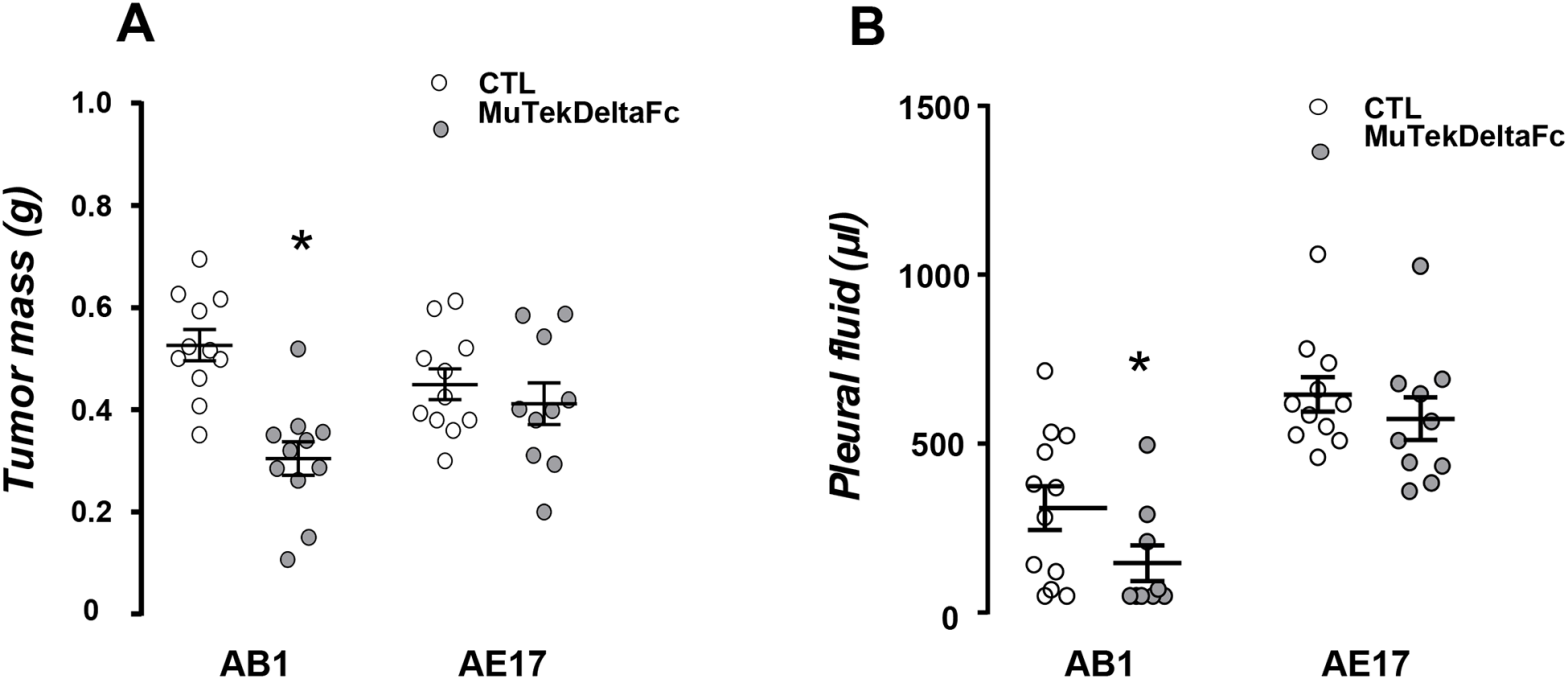
Murine Tek-Delta Fc abrogates AB1 mesothelioma progression AB1 and AE17 cells were intrapleurally injected into syngeneic Balb/c and C57Bl/6 mice respectively. Mice were intraperitoneally administered with MuTekDeltaFc 40 mg/kg (body weight) or vehicle thrice per week. Fourteen days later mice were sacrificed and mesothelioma tumors were excised and weighed **(A)** and pleural fluid was retrieved and quantified **(B)**. Data presented as mean±SEM, n=10-13, ^*^p<0.05 compared to CTL.

**Figure 4:**
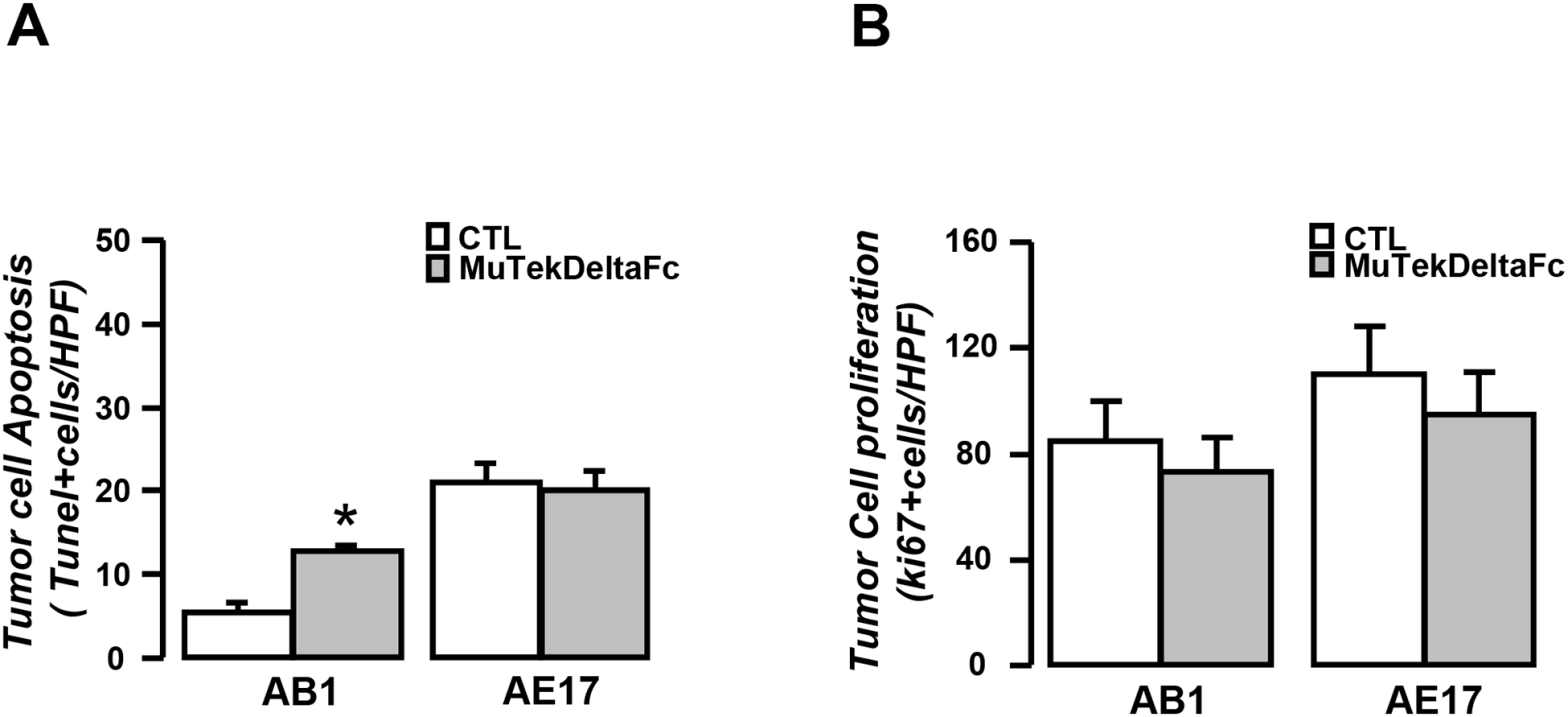
Murine Tek-Delta Fc promotes AB1 mesothelioma cell apoptosis *in vivo* Τumor tissue sections from vehicle (CTL) or MuTekDeltaFc treated animals were analyzed for apoptosis by TUNEL assay **(A)** while tumor cell proliferation rate was evaluated by Ki67 staining **(B)**. Data presented as mean±SEM, n=10-13, ^*^p<0.05 compared to CTL. HPF: High Power Field.

Systemic administration of Murine Tek-deltaFc differentially affected mesothelioma progression in the two models examined. The inhibitor significantly impeded tumor growth and pleural fluid accumulation in Balb/c but not in C57BL/6 tumor model (Figure 3A and 3B). Murine Tek-deltaFc-treated AB1 but not AE17 tumors presented higher apoptosis rates as compared to controls (Figure 4A). Treatment did not impact tumor cell proliferation *in vivo* in either AB1 or AE17 model (Figure 4B). Although both mesothelioma cell lines express Tie-2, treatment with Ang-1, Ang-2 or Murine Tek-deltaFc had no direct *in vitro* effect on tumor cell viability in either (data not shown).

### Murine Tek-deltaFc differentially affects tumor vessel network of mesothelioma tumors

**Figure 5:**
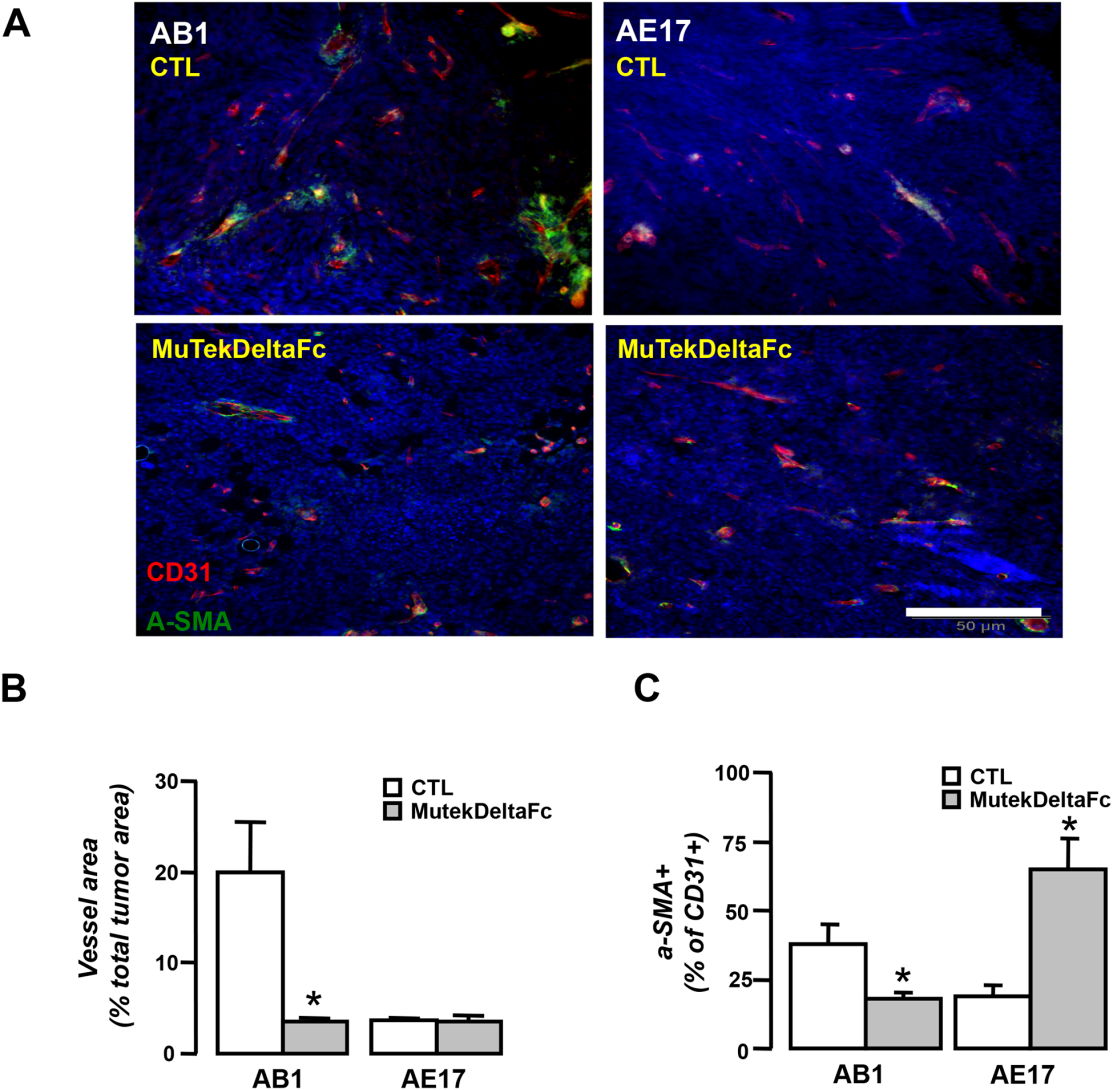
Divergent effects of Murine Tek-Delta Fc on AB1 and AE17 tumor vascular network Tumor tissue cryosections from vehicle (CTL) or MuTekDeltaFc treated animals were analyzed for the expression of CD31 (red) and a-SMA (green) by immunofluorescence staining. **(A)** Representative images. Vessel networks were analyzed for **(B)** proportional vessel area and **(C)** a-SMA+ vessels. Data presented as mean±SEM, n=5-7, ^*^p<0.05 compared to vehicle.

**Figure 6:**
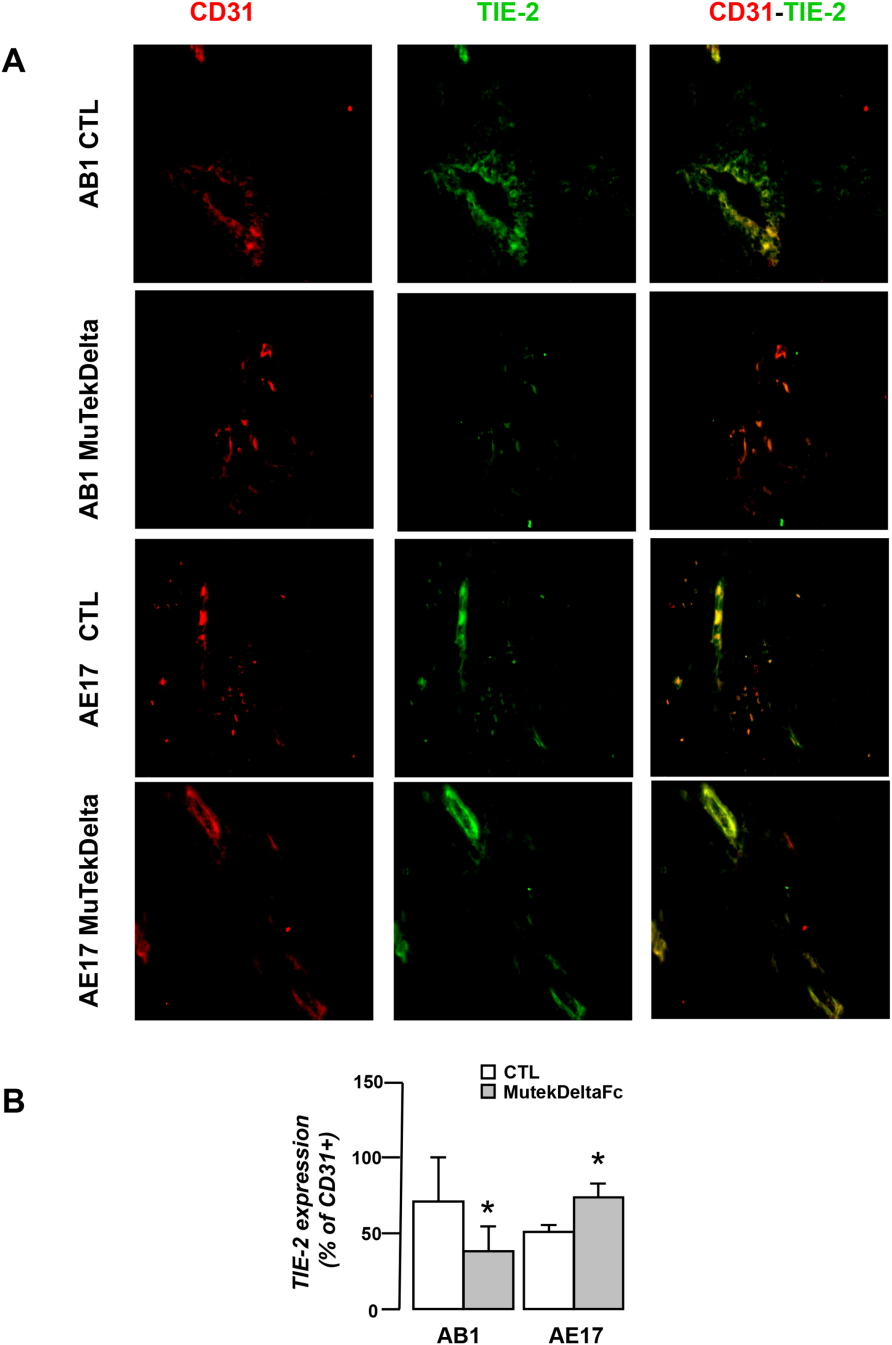
Murine Tek-Delta Fc differentially affects Tie-2 expression by AB1 and AE17 tumor vasculature Tumor tissue cryosections from vehicle (CTL) or MuTekDeltaFc treated animals were analyzed for CD31 (red) and Tie-2 (green) by immunofluorescence staining. **(A)** Representative images **(B)** Vessel networks were analyzed for Tie-2 expression. Data presented as mean±SEM, n=5-7, ^*^p<0.05 compared to vehicle.

We subsequently investigated whether angiopoietin blockade modulates key structural features of tumor vessel networks of mesotheliomas. We observed that AB1 and AE17 tumors not only differ in terms of their baseline vascular profile (Figure 2C/2D) but also that their vascular response to Murine Tek-deltaFc treatment was substantially different. In detail, the inhibitor significantly reduced total vascular area (Figure 5A/5B) and attenuated vessel pericyte coverage and endothelial Tie-2 expression (both markers of vessel stability/maturation, 20) in AB1 tumors (Figure 5A/5C, Figure 6). In contrast, in AE17 tumors, Murine Tek-deltaFc treatment did not affect vascularity, vessel pericyte coverage and endothelial Tie-2 expression (Figure 5C/ Figure 6). Activated and total Tie-2 expression levels were similar among groups (Data not shown).

### Murine Tec-deltaFc affects tumor angiopoietin levels without affecting VEGF

**Figure 7:**
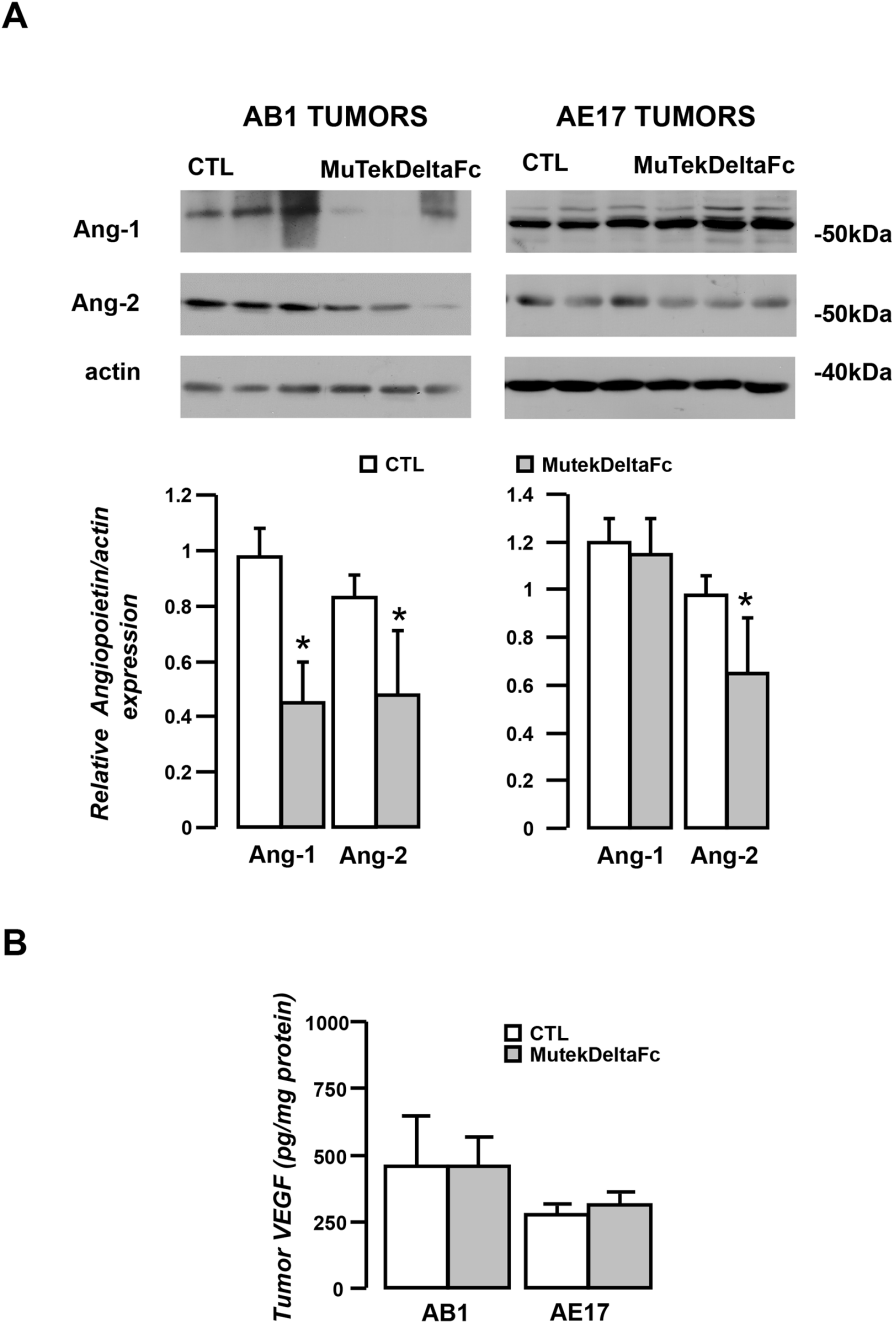
Effects of Murine Tek-Delta Fc in tumor content of angiopoietins and VEGF Tumor tissue lysates of vehicle (CTL) or MuTekDeltaFc treated animals were analyzed by western blotting for the presence of Ang-1 and Ang-2. Results were normalized to actin (**A**, upper panel) Representative blots. (A, lower panel) Densitometric analysis. **(B)** VEGF was also quantified in tumor lysates of vehicle or MuTekDeltaFc treated animals. Data presented as mean±SEM, n=10-13, ^*^p<0.05 compared to vehicle.

Attempting to explain the divergent vascular effects of the inhibitor in the two mesothelioma models we assumed that it might differentially blocked angiopoietin binding to Tie-2 in tumors. We therefore quantified tumor Ang-1 and Ang-2 levels using immunoblotting. It should be emphasized that the size of the angiopoietin bands detected by western blot corresponds to the free (not bound to the s-Tie-2) and therefore active, fraction of Ang-1 and Ang-2, since angiopoietin/s-Tie-2 complexes are expected to have much higher molecular weight. Therefore, treatment-induced changes on free angiopoietin content of the tumor can be used as a surrogate for the level of Ang-1 and Ang-2 neutralization *in vivo*. Active fraction of both angiopoietins was significantly reduced in the AB1 mesotheliomas of the treated animals (Figure 7A). However, in AE17 mesotheliomas, treatment with the inhibitor significantly reduced active Ang-2 but not active Ang-1 (Figure 7A). VEGF was subsequently quantified in tumor lysates to trace the possible stimulation of a compensatory pro-angiogenic signaling. Tumor tissue VEGF levels were not altered in either of the models (Figure 7B).

### Angiopoeitins -1 and -2 are abundant in tumors and pleural fluid of mesothelioma patients

**Figure 8:**
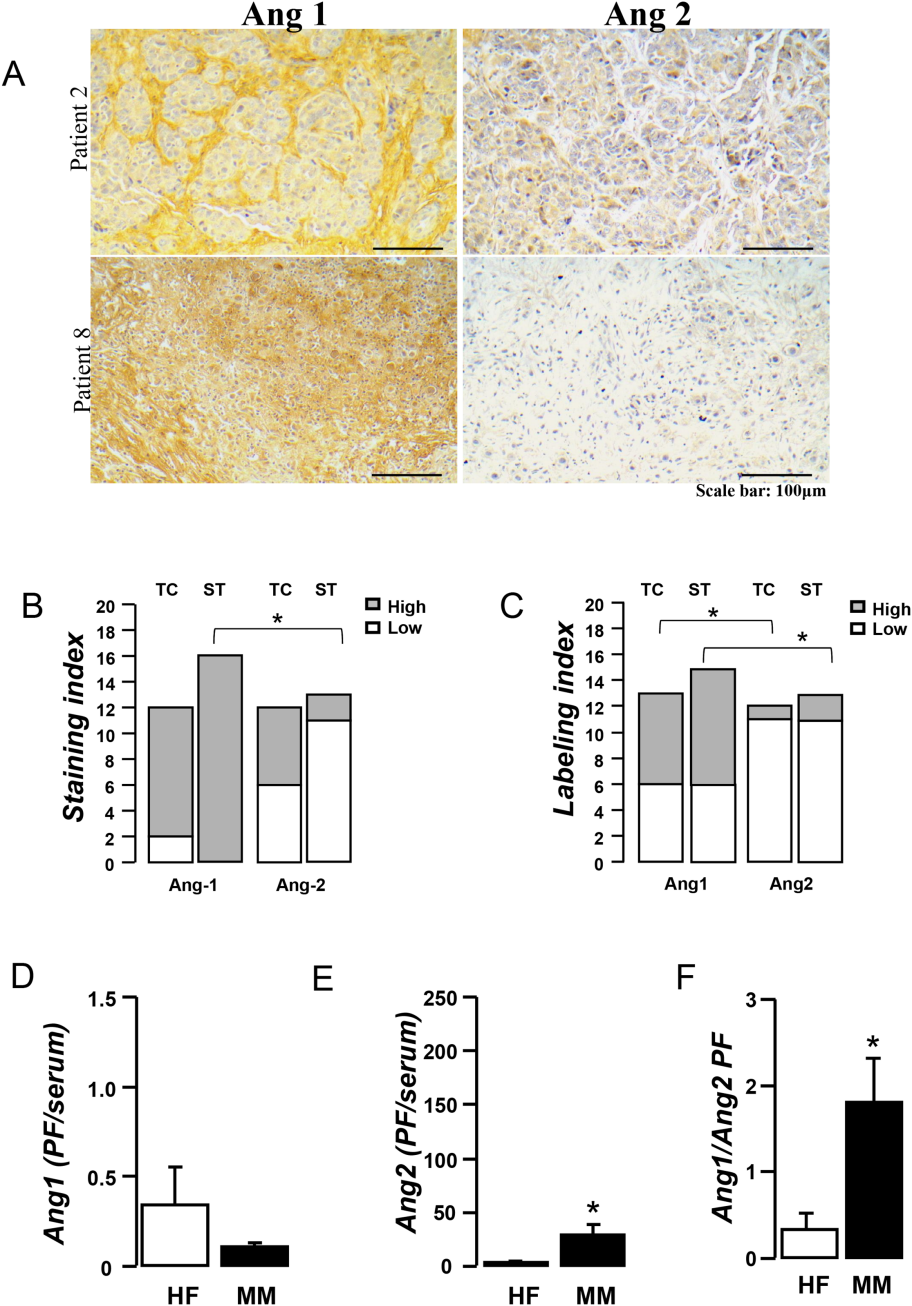
Angiopoietins are abundant in mesothelioma tumors and associated-pleural fluid Tumor tissue sections of 14 mesothelioma patients were stained for Ang-1 or Ang-2 and evaluated by an experienced pathologist. Representative pictures **(A)**. Staining **(B)** (Low: 0-1, High: 2-3) and Labeling **(C)** (Low: 0-2, High: 3-4) indexes of angiopoietins were evaluated in the tumor cell (TC) and stromal (ST) areas of tumor sections. Data presented as mean±SEM, n=14, ^*^p<0.05 from Ang2. Pleural fluid and serum Ang-1 and Ang-2 levels were determined in 14 mesothelioma patients and 16 heart failure patients using ELISA. Serum to pleural fluid levels of Ang-1 **(D)**, Ang-2 **(E)** as well as Ang-1/Ang-2 ratio **(F)** was calculated. Data presented as mean±SEM, n=14-16, ^*^p<0.05 compared to heart failure.

In order to probe the clinical relevance of angiopoietin targeting in mesothelioma we evaluated Ang-1 and Ang-2 content in tumor, pleural fluid and serum of mesothelioma patients. Although Angiopoietins were equally expressed by tumor cells (Figure 8B), Ang-1 was found to be expressed by a larger proportion of tumor cells (Figure 8C). Ang-1 was also found to prevail in the stroma (Figure 8B and Table 1). Ang-1 outbalanced Ang-2 in the pleural fluid (Figure 8F). Nevertheless, Ang-2 pleural fluid levels were higher than the corresponding serum levels (Figure 8E) and Ang-1 (Figure 8D) serum levels were higher than the corresponding pleural fluid ones in every patient with MPM. The above suggest that both angiopoietins are up-regulated in mesothelioma rendering them attractive targets for mesothelioma treatment.

**Table 1:** IHC staining evaluation of Ang-1 and Ang-2 in mesothelioma patients

Case	Ang 1 SI^1^ × LI^2^ Tumor cells	Ang 2 SI × LI Tumor cells	Ang 1 SI × LI stroma	Ang 2 SI × LI stroma
Patient 1	2 × 3	2 × 1	2 × 2	1 × 1
Patient 2	2 × 1	2 × 2	3 × 4	1 × 2
Patient 3	2 × 2	Non Available	2 × 3	Non Available
Patient 4	2 × 3	2 × 1	2 × 2	2 × 1
Patient 5	3 × 1	1 × 1	3 × 2	1 × 2
Patient 6	[-]	[-]	3 × 3	1 × 2
Patient 7	1 × 4	1 × 1	2 × 3	1 × 1
Patient 8	2 × 4	2 × 1	3 × 3	1 × 2
Patient 9	2 × 2	1 × 2	2 × 3	1 × 3
Patient 10	2 × 4	2 × 2	2 × 4	1 × 2
Patient 11	2 × 2	1 × 1	2 × 2	1 × 1
Patient 12	2 × 3	2 × 1	2 × 3	2 × 2
Patient 13	2 × 2	2 × 2	2 × 2	2 × 2
Patient 14	1 × 4	0	3 × 2	1 × 1

Tumor tissue sections of 14 mesothelioma patients were stained for Ang-1 or Ang-2 and evaluated by an experienced pathologist.

1 Staining index (SI) 0: no 1: faint 2: moderate 3: intense 2 Labeling index (LI) 0: no staining 1: 1-25% 2: 26-50% 3: 51-75% 4: 76-100%.

## DISCUSSION

The present study aimed to unveil the impact of Murine Tek-deltaFc, a soluble-Τie2 that inhibits the interaction of the naturally occurring receptor with Ang-1 and Ang-2, in mesothelioma progression *in vivo*. We used pleural AB1 and AE17 mesothelioma models and demonstrated that: a. Murine Tek-deltaFc limited AB1 but not AE17 mesothelioma growth *in vivo* whereas it did not affect the viability of any of them *in vitro*. b. Murine Tek-deltaFc promoted tumor cell apoptosis *in vivo* and impaired tumor angiogenesis in AB1 but not in AE17 mesotheliomas. c. AB1 tumors that responded to the treatment were more vascularized and displayed higher endothelial Tie-2 expression than the non-responding AE17 tumors. d. Albeit Murine Tek-deltaFc significantly reduced the levels of active (s-Tie-2-unbound) Ang-2 in both AB1 and AE17 tumors, active tumor Ang-1 levels were substantially reduced only in the AB1 tumors. e. Both Angiopoietins (though mostly Ang-1) are vastly evident in tumor tissues and pleural fluid of mesothelioma patients.

This is the first study investigating the effect of systemic administration of an angiopoietin inhibitor in mesothelioma. We have previously tested this agent in adenocarcinoma-induced experimental malignant pleural effusion, where it was shown to abrogate pleural fluid accumulation and tumor dissemination [17]. Furthermore, Murine Tek-deltaFc has also been successfully tested in colorectal carcinoma xenographs [18]. In agreement with previous observations [17, 18], the anti-mesothelioma properties of Murine Tek-deltaFc should be mainly ascribed to its anti-angiogenic potential: First, in contrast to a previous study, demonstrating that Ang-1 promotes proliferation of Tie-2 (+) mesothelioma cells [15], we found that neither angiopoietins nor the inhibitor impacted the *in vitro* growth of the cells in our study, even though they both express active Tie-2. This implies that Ang-1-stimulated proliferation is not a universal property of all Tie-2 expressing mesotheliomas. Secondly, Murine Tek-deltaFc limited tumor cell survival and tumor growth *in vivo*, only in the model in which it succeeded in blocking tumor angiogenesis, suggesting that the observed mesothelioma cell apoptosis most likely is a result of tumor blood supply restriction. An important question then arises: how can one explain the divergent response of tumor vasculature to the Murine Tek-deltaFc between AB1 and AE17 tumors? Is it attributed to different baseline expression of Tie-2 or to the angiopoietins by the tumor, or even to the anatomical/functional features of the tumor vessel network? In an effort to unravel this issue, we found that the inhibitor-responding AB1 tumors were more vascular, less hypoxic and presented higher endothelial Tie-2 expression compared to the non-responding AE17 tumors. In other words, Murine Tek-deltaFc targets are more abundant and may be more functionally important in AB1 tumors than in AE17 ones. Moreover, the observed divergence of the vascular phenotype and hypoxic profile between the two models suggests higher dependence of AB1 tumors on blood supply (therefore more vulnerable to anti-angiogenic agents) and/or increased resilience of AE17 tumors to hypoxia (therefore more resistant to anti-angiogenic treatment). In addition, higher endothelial Tie-2 expression may be indicative of a greater reliance on Tie-2 signaling for AB1 tumor angiogenesis which implies a greater sensitivity to Murine Tek-deltaFc treatment. Similarly, Fathers et al, [18] previously reported that Murine Tek-deltaFc anti-angiogenic activity comes in parallel to the abundance of Tie-2 expressing tumor vessels. In addition to the differences in the base-line features of tumor vasculature and given that Murine Tek-deltaFc prevents Tie-2 binding with its ligands, diverse tumor angiopoietin blocking should be also considered as a possible explanation for the observed contrasting activity of the inhibitor between the two models. Relative to this, AB1 mesotheliomas contains more Ang-2 and less Ang-1 than AE17 ones. Murine Tek-deltaFc conferred a significant reduction of active Ang-2 tumor levels in both models, while active Ang-1 was significantly reduced only in the AB1 model. Binding IC_50_ concentrations for Murine Tek-deltaFc to Ang-1 and Ang2 are similar (14 nM and 10 nM, respectively) [21]. Inadequate Ang-1 neutralization in the AE17 model *in vivo* can thus be attributed to the high baseline levels of tumor-derived Ang-1. Excessive post-treatment unbound and therefore, active Ang-1 along with mostly neutralized Ang-2 probably explains the treatment-enhanced vessel pericyte coverage (a process promoted by Ang-1 and inhibited by Ang-2) [14] in AE17 tumors. It should be noted that pericytes, apart from acting as physiological vessel stabilizers can be also recruited by the tumors to serve as a protective cloak that silences the anti-angiogenic signals to endothelial cells [22]. Summing up, Murine Tek-deltaFc treatment can curtail the growth of highly vascularized and endothelial Tie-2 expressing mesotheliomas while its activity is also linked to the tumor content of Ang-1.

From a clinical point of view, our observations greatly support the optimism raised by the positive findings of the recent trial using the anti-VEGF antibody [9] that entails that anti-angiogenic therapy can be a viable strategy for MPM and pave the way for clinical investigation of anti-angiopoietin treatment in mesothelioma. This is further supported by the observation that both angiopoietins are abundant in human mesotheliomas and mesothelioma-related pleural effusions. Moreover, the human analogue of Murine Tek-deltaFc, AMG-386, which has been tested in various malignancies demonstrated mild toxicity profile and it has so far proven clinically beneficial in ovarian cancer [19, 20, 10] making clinical testing of anti-angiopoietin therapies easier to pursue. Similarly, future clinical trials will examine the possibility that tumor vascularity, endothelial Tie-2 expression and tumor cell Ang-1 expression can serve as markers that can predict response to treatment, as indicated by our preclinical studies presented here. This possibility can be also investigated retrospectively using archival material from clinical studies conducted with the human analogue of Murine Tek-deltaFc). Another clinically relevant observation of the present study is that tumor VEGF levels were not altered by the treatment. This demonstrates that angiogenesis blockade by an anti-angiopoietin agent may cause no compensatory proangiogenic signaling and therefore treatment resistance, resulting to a major disadvantage of most VEGF-targeting antiangiogenic agents [22]. We believe that all the above strongly support the notion of clinical testing of angiopoietin blockage in mesothelioma.

In conclusion, preclinical evaluation of Murine Tec-deltaFc has highlighted the central role of angiopoietin/Tie-2 axis in mesothelioma angiogenesis and growth and paves the way for clinical testing of anti-angiopoietin interventions in mesothelioma patients. Tumor vascularity, endothelial Tie-2 expression and tumor Ang-1 expression could be investigated as markers predicting patient response.

## MATERIALS AND METHODS

### *In vitro* studies

AE17 and AB1 murine mesothelioma cells [23, 24] were kindly provided by Dr YCG Lee, Perth, Western Australia and maintained as previously described [16]. Both cell lines used in the present study were periodically monitored for mycoplasma presence by PCR. Their morphology was examined on a regular basis. To investigate whether angiopoietins or the inhibitor, impact tumor cell growth, 3x10^3^ tumor cells were seeded on 96-well plates and 24h later were treated with 300-1000 ng/ml Ang-1 or Ang-2 and 1-100 μg/ml Murine Tek-deltaFc or vehicle for 24h. Cell viability was subsequently evaluated by MTS reduction (Promega, Madison, WI).

### *In vivo* studies

Mice were purchased from BSRC Al. Fleming (Vari, Greece) and were housed at the Animal Model Research Unit of Evangelismos Hospital, (Athens, Greece) receiving food and water ad libitum. Experiments were approved by the Veterinary Administration Bureau, Prefecture of Athens, Greece under compliance to the national law and the EU Directives. Murine Tek-deltaFc was kindly provided to us by Amgen (Amgen Inc., Thousand Oaks, CA). AE17 or AB1 (5×105) mesothelioma cells were intrapleurally injected to 8-10 week-old male C57BL/6 or Balb/c syngeneic mice, respectively. Four days upon tumor cell implantation, when pleural tumors are already evident [16] animals were divided into two groups, receiving either Murine Tek-deltaFc or vehicle. Treatment was intraperitoneally administered at 40 mg/kg body weight thrice per week. Animals were euthanized 14 days after pleural delivery of tumor cells. Pleural fluid, tumors, lungs and blood were collected and stored for subsequent analysis. Pleural fluid was retrieved and quantified; mesothelioma tumors were collected and weighed.

### Immunohistochemistry and immunofluorescence

Tumor tissue paraffin sections were immunohistochemically analyzed for Ki-67 (clone D385, Cell Signaling, Danvers, MA) for evaluation of tumor cell proliferation. Tumor cell apoptosis was estimated by TUNEL as previously described [25]. For immunofluorescence analysis, tumor sections were fixed and stained for the presence of CD31 (clone MEC 13.3, BD Biosciences, Athens, Greece) and a-SMA (clone 1A4, Sigma-Aldrich, Steinheim, Germany) for endothelial and pericyte staining, respectively. Alternatively, tumor sections were stained for Tie-2 (CL16) and CD31 presence (Abcam, Cambridge, UK) in order to evaluate the Tie-2 expressing vessels. Vessel networks were subsequently analyzed for vessel area, pericyte area and Tie-2 distribution using ImageJ software (National Institutes of Health, Bethesda, MD). Visualization and quantification of hypoxic areas of tumors was performed upon immunofluorescent detection of pimonidazole adducts. Briefly, mice bearing AB1 or AE17 mesothelioma tumors were injected with 60mg/kg body weight pimonidazole and sacrificed 90 min later. Mesothelioma tumors of relatively equal size were cryopreserved and subsequently stained with anti-pimonidazole adducts antibody conjugated to Dylight549 (Hypoxyprobe kit, Chemicon International, Inc, Temecula, CA). Tumor hypoxia was evaluated under a fluorescent microscope and quantified using ImageJ software (National Institutes of Health, Bethesda, MD).

### Quantification of angiogenesis related factors

Angiopoietin (-1 and -2) levels were determined in mesothelioma cells, pleural tissues and tumor lysates by western blot. Murine Ang-1 and Ang-2 mRNA levels were subsequently evaluated in reference to GAPDH expression by Real-time PCR using the following primers: Ang-1 FOR:CCATGCTTGAGATAGGAACCAG, Ang-1 REV:TTCAAGTCGGGATGTTTGATTT, Ang-2 FOR:AGCAGATTTTGGATCAGACCAG Ang-2 REV:GCTCCTTCATGGACTGTAGCTG, mGAPDH FOR:AGGTCGGTGTGAACGGATTTG and mGAPDH REV:TGTAGAACCATGTAGTTGAGGTCA.

Tie-2 activation levels in tumor cells and tumor lysates were determined by immunoprecipitation and normalised to total Tie-2 levels. VEGF concentration was quantified in cell supernatants and tumor lysates by ELISA (Peprotech, Rocky Hill, NJ)

### Human studies

Studies using human samples were approved by the ethics committee of the Evangelismos Hospital. Paraffin blocks of tumor tissue from 14 mesothelioma patients (71.7+/-2.8 years of age, 5:2 male:female ratio, all epitheliod subtype) were obtained from the archive of the Department of Anatomic Pathology, Evangelismos Hospital, Greece. Mesothelioma tumor tissue sections were stained for Ang-1 (ab8451) and -2 (ab153934) (Abcam, Cambridge, UK) and staining was evaluated by an experienced pathologist. Staining of angiopoietins was assessed in a semi-quantitative manner as previously described [26]. Tumor cell and stromal compartments of tissue sections were independently evaluated and each section was assigned two scores: one describing its staining intensity (0, no staining; 1, weak staining; 2, moderate staining; and 3, intense staining) and another referring to the proportion of cells stained (0, no cells staining; 1, 1–25%; 2, 26–50%; 3, 51–75%; 4, 76–100%).

Pleural fluid and serum samples of 14 mesothelioma (62+/-1.7years of age, 6:1 male:female ratio) and 16 heart failure patients (77+/-5,2 years of age, 13:3 male:female ratio) were retrieved from our bank under all patients' informed consent [27]. Ang-1 and -2 levels in fluids and matching sera were measured by ELISA (R&D systems, Minneapolis, MN)

### Statistics

All values are presented as mean ± standard error of mean (SEM). Differences between groups were evaluated using the Student’s t-test with least square difference post-hoc tests, as appropriate. Log rank Chi square test was used to determine the differences of Angiopoietin intensity and staining indexes in mesothelioma tumors. P values < 0.05 were considered significant. Statistical analysis was performed using the Statistical Package for the Social Sciences v.13.0.0 (IMB, Armonk, NY).
